# Efficacy and Safety of Topical Application of Plant‐Based Products on Skin Aging in Healthy Individuals: A Systematic Review and Meta‐Analysis of Randomized Controlled Trials

**DOI:** 10.1111/jocd.16710

**Published:** 2024-12-09

**Authors:** Fengrui Cheng, Jinhong Feng, Ziling Cao, Qu Duan, Haoying Li

**Affiliations:** ^1^ Hospital of Chengdu University of Traditional Chinese Medicine Chengdu Sichuan China; ^2^ Clinical Medical College Chengdu University of Traditional Chinese Medicine Chengdu Sichuan China; ^3^ Hospital of Baoji Traditional Chinese Medicine Baoji Sichuan China

**Keywords:** meta‐analysis, plant extract, skin aging, systematic evaluation, topical treatment

## Abstract

**Background:**

Recently, numerous topical products containing plant‐based ingredients have been reported to resist skin aging. However, there is a lack of sufficient evidence to substantiate these claims. This paper presents a comprehensive review and meta‐analysis to evaluate the efficacy and safety of topical products containing plants or plant extracts in anti‐aging.

**Methods:**

Four databases—PubMed, Embase, Web of Science, and the Cochrane Library (CENTRAL)—were systematically searched for articles related to plant‐based interventions and skin aging, covering the period from January 2000 to December 2024. A total of eight randomized controlled trials (RCTs) met the inclusion criteria and were included in the meta‐analysis.

**Results:**

Products containing plant extracts demonstrated a statistically significant difference in improving skin hydration and skin elasticity, reducing melanin and erythema compared to other products. No significant statistical difference was observed in reducing transepidermal water loss (TEWL). Subgroup analysis revealed a significant statistical difference in improvement overall skin elasticity (R2) during short‐term (≤ 8 weeks)treatments, while no statistical difference was observed during long‐term (> 8 weeks)treatments. Additionally, no significant difference was observed in the specific measurements of skin elasticity, including R5 (net elasticity) and R7 (the ratio of elastic recovery to total deformation). Regarding safety, no adverse events were reported in six studies, while the remaining two studies did not specify whether adverse events occurred.

**Conclusion:**

The meta‐analysis results indicated that botanical preparations significantly enhanced skin hydration, reduced melanin levels and erythema, and increased overall skin elasticity. However, the analysis did not provide sufficient evidence to support a reduction in transepidermal water loss (TEWL), or improvements in R5 (net elasticity) and R7 (the ratio of elastic recovery to total deformation).

**Systematic Review Registration:**

PROSPERO (york.ac.uk) identifier: CRD42023478803

## 1 Introduction

Baby‐soft skin has long been considered the ideal skin type. However, both external factors, such as environmental pollution [1], and ultraviolet radiation [2], and intrinsic factors including genetics [3] and hormones [4], significantly contribute to skin aging. The process of skin aging not only diminishes the skin's aesthetic appeal but also impairs wound healing, increases the risk of infections, and contributes to various dermatological disorders [5, 6]. It is widely acknowledged that the primary mechanisms of skin aging include inflammation [7], autophagy, oxidative stress [8], DNA damage [9], advanced glycation end‐products (AGEs) [10], and disruptions to the biological clock [11, 12]. The generation of reactive oxygen species (ROS) caused by skin exposure to ultraviolet light triggers the release of inflammatory cytokines and leads to skin dryness, decreased elasticity, irregular pigmentation, and skin erythema [13, 14]. Skin hydration refers to the ability of the outermost layer of the skin to bind water, while transepidermal water loss refers to the extent to which water evaporates and diffuses from deeper layers of the skin, both of which are important factors affecting the skin barrier and causing skin dryness [15, 16]. The damage caused by skin aging process to dermal connective tissues (collagen, elastic fibers, and proteoglycans) manifests as a reduction in skin elasticity [17]. Erythema is primarily associated with inflammation resulting from prolonged exposure to UVA [18]. UVB irradiation stimulates melanin production in melanocytes, which is accompanied by the activation of tyrosinase [19]. New substances and medications targeting various manifestations of skin aging are increasingly being introduced to the market. However, their topical application raises considerable safety concerns, potentially causing adverse reactions such as peeling, redness, and itching [20, 21, 22]. Therefore, it is essential to develop safer and more effective topical products for the prevention of skin aging.

Plant extracts can be derived from either the entire plant or specific parts, such as fruits, leaves, or other components [23]. Primary metabolites are generally defined as the three essential nutrients required for a daily diet—sugars, fats, and proteins. Terpenes and phenolics are examples of secondary metabolites that function as immunomodulatory, antimicrobial, and anti‐inflammatory agents in treating common skin conditions [24, 25]. Botanicals have been incorporated into skincare products for thousands of years, and their use in anti‐aging cosmetics continues to grow [26]. Numerous clinical studies have explored the role of plants in maintaining skin health; however, flaws in the design methodologies of some studies have been noted [27, 28]. Therefore, this literature has been systematically evaluated, and a meta‐analysis was conducted through a comprehensive search of randomized controlled studies on the anti‐aging effects of plants and plant extracts. The objective was to assess the efficacy and safety of topical botanical products in the aging process of healthy individuals' skin and to provide a scientific foundation for routine skincare practices.

## 2 Method

This study adheres to the PRISMA (Preferred Reporting Items for Systematic Reviews and Meta‐Analyses) guidelines, ensuring rigorous criteria for inclusion, eligibility, and selection [29].

### 2.1 Search Strategy

The systematic search was independently conducted by three reviewers across PubMed, Embase, Web of Science, and the Cochrane Library, covering studies published from January 2000 to 2024 relevant to the topic. The primary search terms included: (1) plant or plant extract derivatives, and (2) skin aging or skincare. Adjustments were made to account for the variation in MeSH terms across databases, with detailed search queries for each database provided in the Supporting Information.

### 2.2 Eligibility Criteria

The selected studies were required to meet the following criteria:

(1) Type of research: Parallel randomized controlled trials evaluating the effects of plants or plant extracts on human skin aging, with outcomes including melanin levels, erythema, skin hydration, skin elasticity, or transepidermal water loss (TEWL). Only studies with two or more reports of the same outcome measures for skin aging were included.

(2) Subjects of research: Healthy individuals aged 15–65 years, with skin problems, such as facial wrinkles, lack of skin elasticity, dull complexion, eyelid bags or dark circles, or skin pigmentation. The exclusion criteria were as follows: (a) pregnancy, breastfeeding, or having metabolic diseases such as diabetes mellitus; (b) presence of atopic dermatitis, acne, or other inflammatory skin diseases.

(3) Types of intervention: Controlled trials of the topical use of plant products were considered eligible, provided that the same plant or plant extract was not present in the placebo. Oral forms of therapy were excluded.

### 2.3 Study Selection

**FIGURE 1:**
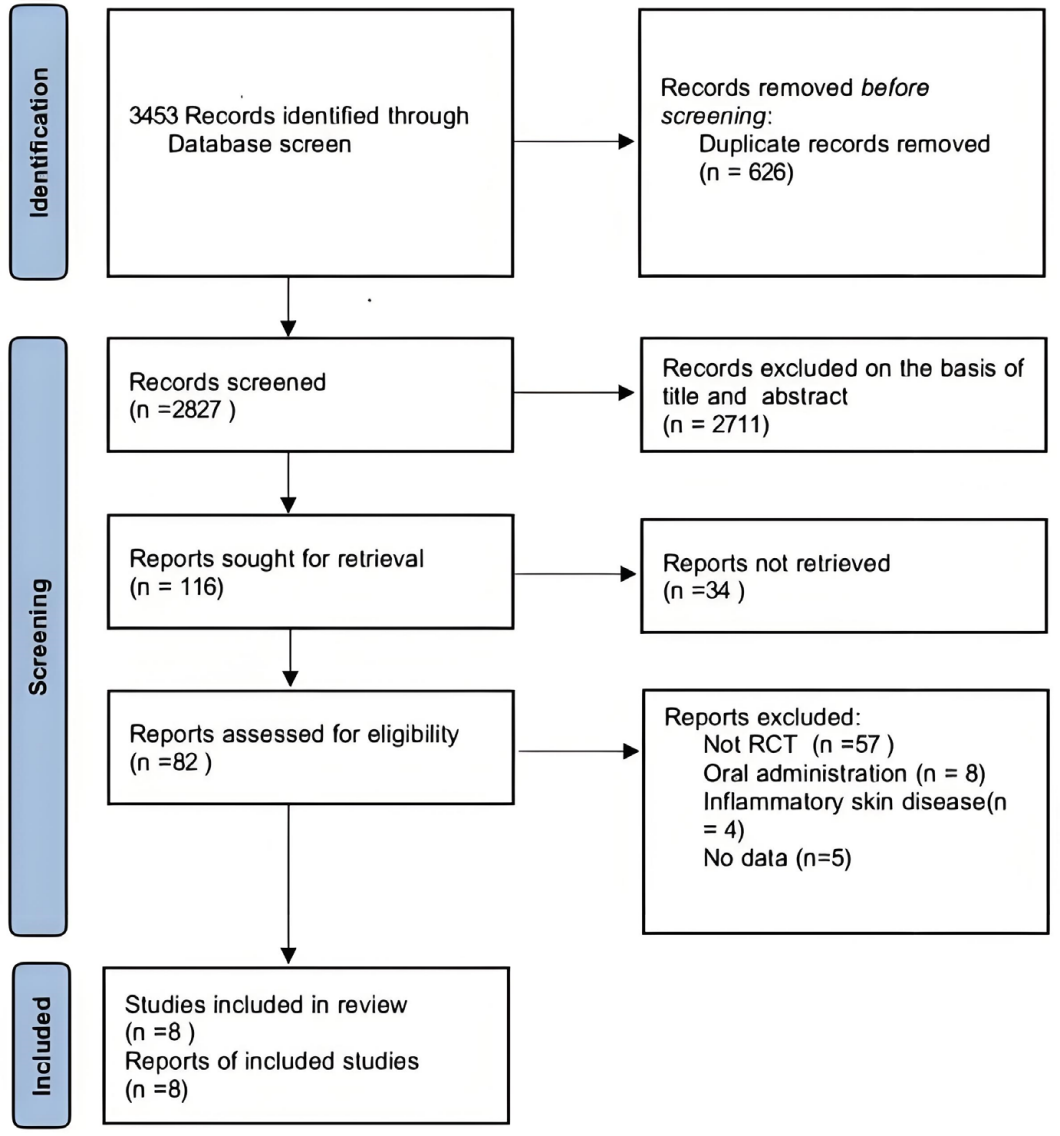
Flow diagram illustrating the study selection process.

The literature retrieved using the specified search terms was independently imported into EndNote X9 by three reviewers. Duplicates were removed using the software, and the reviewers independently screened the titles and abstracts of each study to assess eligibility based on the established criteria. In cases of disagreement, discussions were held with H.L. to reach a consensus (Figure 1).

### 2.4 Data Extraction

The data extracted by the three investigators from each included study are recorded in Table S2. These data include the author(s), country and year of publication, the number of participants, their age (or mean age), and their skin condition, a detailed description of the intervention (including duration and content), and the outcome measures (instruments and sites). The safety of the intervention groups was evaluated based on adverse events (AEs) reported during the intervention. Baseline and endpoint values, along with their standard deviation (SD), were also extracted from the study outcomes, including melanin, erythema, TEWL, R5, and R7. Additionally, baseline values, endpoint values, and variability standard deviations (SDs) in skin hydration and R2 at different intervention times (≤ 8 weeks, > 8 weeks) were also extracted for subgroup analysis.

### 2.5 Quality Assessment

The literature was assessed using the Cochrane Risk of Bias Tool [30], which evaluates seven criteria: selection bias (random sequence generation and allocation concealment), performance bias (blinding of participants and personnel), detection bias (blinding of outcome assessment), attrition bias (incomplete outcome data), reporting bias (selective reporting), and other biases. This assessment classified the studies into “low,” “unclear,” and “high” risk categories, and corresponding graphs were generated with yellow, red, and green percentages. Adhering to the Cochrane Collaboration's standards, the three authors carefully reviewed the entire text and independently evaluated each document's risk level. In cases of disagreement, the team discussed the issues collectively and made a final decision in consultation with.

### 2.6 Statistical Analysis

An independent meta‐analysis was conducted using RevMan (version 5.3) to assess the overall effect size of each of the eight included studies. Subgroup analyses were performed to evaluate the effects of different treatment durations on skin hydration and overall skin elasticity.

The standardized mean differences (SMDs) for continuous variables were independently calculated for each group using a random‐effects model. The mean difference was determined by subtracting the baseline value from the endpoint value. If the change in standardized mean differences (SMDs) relative to the baseline was not reported, the SMD value could be computed using a formula based on the baseline and endpoint standard deviations (SDs) for both the treatment and placebo groups.

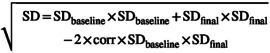




As the baseline‐final correlation coefficients (corr) were not provided in these studies, a corr value of 0.5 was assumed, with a range of 0.4–0.6 based on values reported in most of the literature.

Several studies selected different sites for measurement. When both the palmar and dorsal sides of the hand were measured in one study, we selected the dorsal side because the dominant forearms and palms exhibit the highest values of water loss due to physical, emotional, and thermal mechanisms during perspiration. If a study randomized both a placebo and a plant‐based formulation to the arms of the participants, it was treated as two separate studies. Effect sizes for melanin, erythema volume, transepidermal water loss (TEWL), skin elasticity, and skin hydration were reported as 95% confidence intervals (CIs) around the standardized mean difference (SMD), with *p* > 0.05 not considered statistically significant. This approach was taken because not all studies provided data for these outcomes.

Heterogeneity was assessed using the Higgins *I* ^2^ (*I* ^2^) statistic, as illustrated in the legends accompanying each forest plot. An *I* ^2^value of 0% indicates that all observed variation in effect sizes is attributable to sampling error, whereas an *I* ^2^ value of 100% suggests that all variation is due to true differences in effect sizes across studies [31]. The interpretation of *I* ^2^ values is as follows:

*I* ^2^ = 0% − 25%: low heterogeneity.

*I* ^2^ = 25% − 50%: medium heterogeneity.

*I* ^2^ = 50% − 75%: high heterogeneity.

*I* ^2^ = 75% − 100%: very high heterogeneity.

## 3 Results

### 3.1 Identification of Researches

A total of 3453 papers were retrieved from various databases: 662 from Web of Science, 1013 from Embase, 979 from PubMed, and 799 from the Cochrane Library. After applying the predetermined inclusion and exclusion criteria, eight publications advanced through the screening stages and were included in the meta‐analysis and comprehensive review.

### 3.2 Risk of Bias Within Researches

**FIGURE 2:**
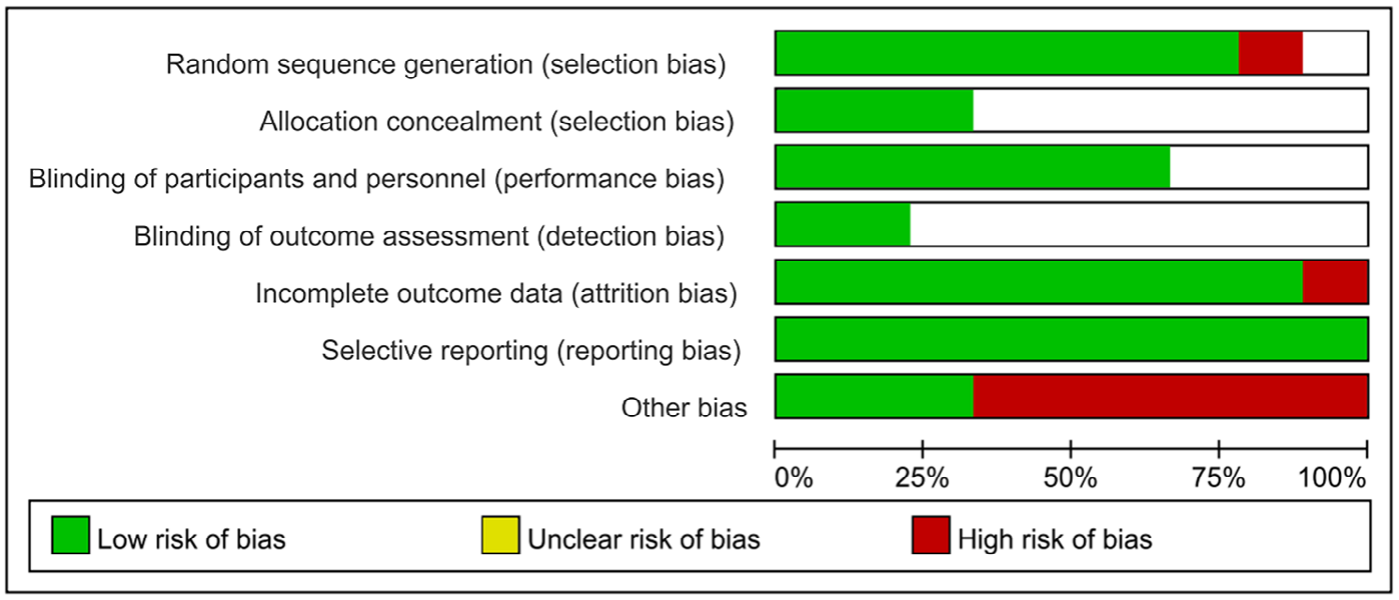
An overview of the review authors' assessments for each risk of bias domain.

The assessment of the risk of bias (ROB) for the eight reviewed papers, expressed as a percentage for each ROB item across the selected studies, is illustrated in Figure 2, based on the researchers' evaluations. The individual assessments for each trial are depicted in Figure S1. Six studies (75%) adequately reported randomization procedures. One study used odd‐even randomization and was therefore evaluated as high risk, and the risk of bias in one study (12%) was unclear. Regarding allocation concealment, only three studies (38%) adequately reported methods such as computer‐generated block randomization tables and randomization codes. Six studies (75%) claimed to be double‐blind, while the remaining studies did not report their blinding status. The blinding of outcome assessment (DB) was generally associated with an unclear risk of bias, with only two studies (25%) identified as low risk. Attrition bias (AB) was primarily assessed as low risk, with only one study classified as high risk due to excessive sample dropout. All eight studies (100%) provided specific data and were therefore evaluated as low risk for reporting bias. “Other sources of bias” primarily involved potential conflicts of interest and insufficient details regarding the physical environmental conditions.

Table S1 provides detailed characteristics of each included study. The eight studies focusing on the topical application of botanicals or plant extracts to mitigate the aging process recruited 396 participants from seven countries. Three studies involved participants from Europe, while the other five included participants from Asia. The studies had a slightly higher proportion of female participants, with ages ranging from 15 to 65 years. The table also includes detailed information on skin characteristics, such as lack of elasticity and dry skin, for each study.

### 3.3 Skin Elasticity

**FIGURE 3:**
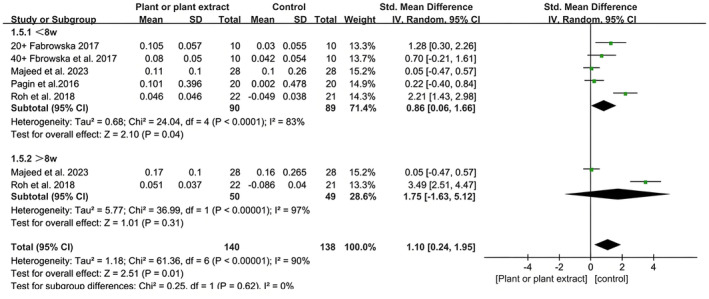
A forest plot derived from a meta‐analysis of these five trials, illustrating the difference in total skin elasticity (R2) between the intervention and placebo groups.

Four studies reported on elasticity outcomes, involving 190 participants in the plant product group and 187 participants in the control group. In one of these studies, plant extracts or placebo were randomly applied to the left and right forearms of the participants [32], which was subsequently treated as two separate studies in this analysis. Two studies used a single plant extract ingredient [33, 34], while the other two utilized complex plant extract formulations [32, 35]. The product type in one study was an emulsion, while the others used creams. The longest study duration was 12 weeks. The MPA 580 instrument was employed in all studies to measure skin elasticity (Figure 3).

The meta‐analysis showed that plants or plant extracts significantly increased skin elasticity compared to placebo for a treatment (SMD 1.10; 95% CI, 0.24–1.95; *p* = 0.01). However, there was no significant difference in skin elasticity with the use of plant products for a treatment duration of > 8 weeks (SMD 1.75; 95% CI, −1.63to 5.12, *p* = 0.31), indicating that the effect sizes did not increase with longer treatment duration. Regarding heterogeneity, the analysis revealed high levels of heterogeneity (*I* ^2^ = 83%, *I* ^2^ = 97%). Notably, the heterogeneity in the ≤ 8 weeks group decreased significantly (*I* ^2^ = 45%). Additionally, the overall effect remained unchanged after excluding the study by Roh from the analysis of total elasticity. This may be attributed to the control group in Roh et al.'s study, which used a mixture of several herbs.

**FIGURE 4:**
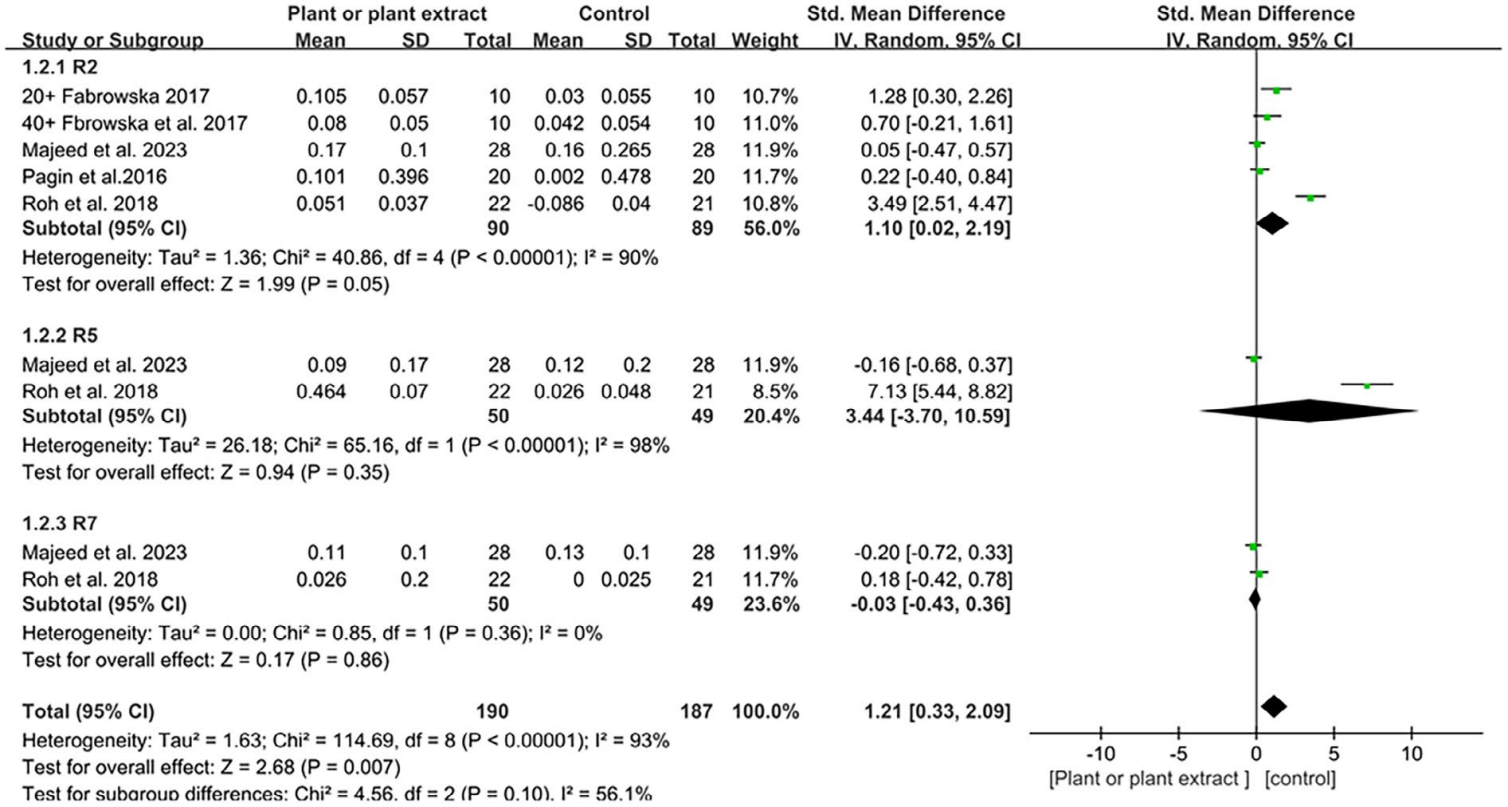
A forest plot derived from a meta‐analysis of these five trials, illustrating the corresponding measurements on skin elasticity.

Figure 4 presents the meta‐analysis of corresponding measurements on skin elasticity: R2 (SMD 1.10; 95% CI, 0.02–2.19, *p* = 0.5), R5 (SMD 3.44; 95% CI, −3.7 to 10.59, *p* = 0.35), and R7 (SMD −0.03, 95% CI, −0.43 to 0.36, *p* = 0.86), which showed no discernible impact of plant‐based products on these skin elasticity parameters.

### 3.4 Skin Hydration

**FIGURE 5:**
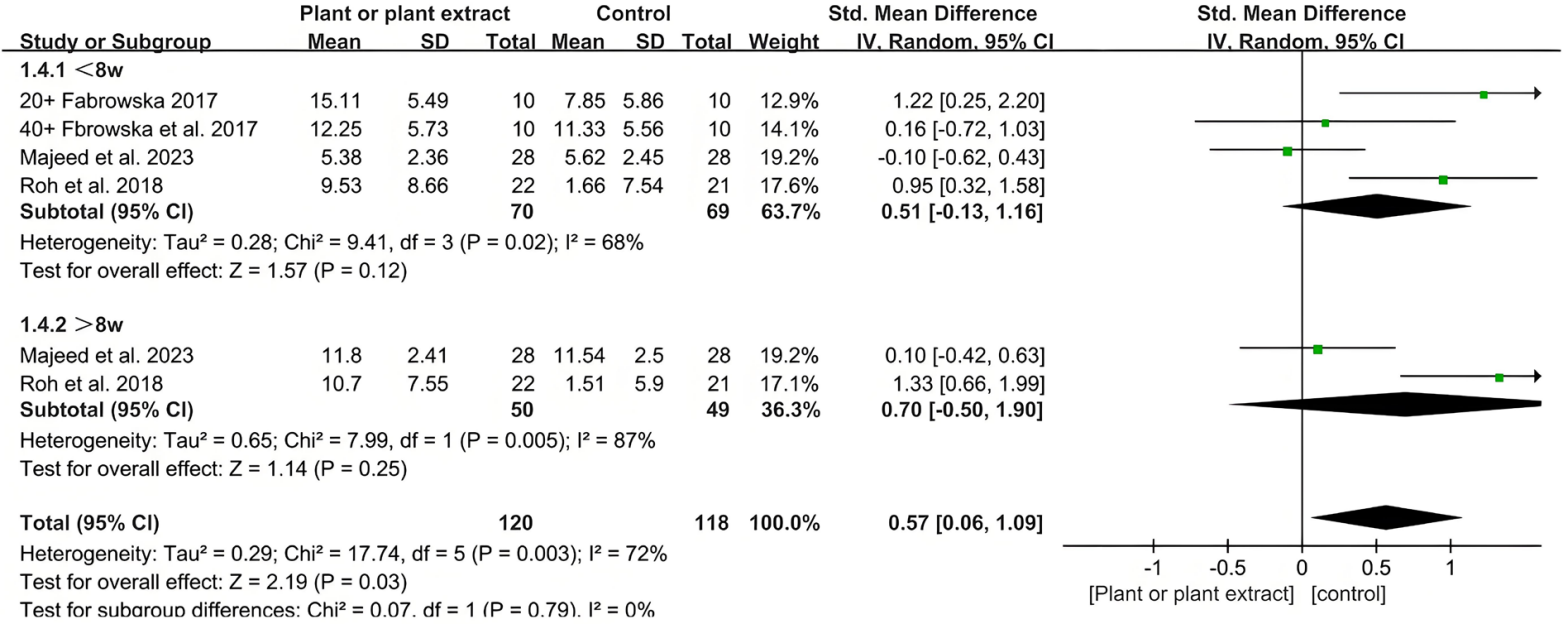
A forest plot derived from a meta‐analysis of these five trials, estimating the difference in skin hydration between the intervention and placebo groups.

Three studies, involving 238 participants from three countries, evaluated skin hydration. In one of these studies, a plant extract and a placebo were randomly applied to the participants' forearms, resulting in the study being treated as two separate trials [32]. The site of measurement in one study was the arm [32], while the other two studies measured on the cheek [33, 35]. The products used in these studies were either emulsions or creams. Skin hydration was measured using the Corneometer CM 825, with intervention durations ranging from 8 to 12 weeks (Figure 5).

The meta‐analysis showed a significant effect of the topical use of botanical products on increasing skin hydration (SMD 0.57; 95% CI, 0.06–1.09, *p* = 0.03). No significant correlation was observed within a treatment duration of ≤ 8 weeks (SMD 0.51; 95% CI, −0.13 to 1.16, *p* = 0.12) or > 8 weeks (SMD 0.70; 95% CI, −0.50 to 1.90, *p* = 0.25). To investigate the reasons for high heterogeneity, each study was excluded individually, revealing that the largest source of heterogeneity was the study by Majeed et al. This may be due to the composition of the placebo products containing other plant extracts, as well as differences in the sources and doses of plant extracts used.

### 3.5 Melanin

**FIGURE 6:**
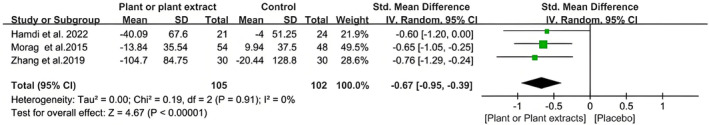
A forest plot derived from a meta‐analysis of these two trials, illustrating the difference in melanin levels between the intervention and placebo groups.

The melanin index was reported in three studies involving 207 participants, all of which used creams as the product type. Two studies utilized single plant extracts [36, 37], while one used a mixture of plant extracts [38]. Melanin levels were measured using the Mexameter on the cheek or periocular area (Figure 6).

The use of plant extracts significantly reduced melanin levels across the three investigations, as indicated by the overall effect size (SMD −0.67; 95% CI, −0.95 to −0.39, *p* < 0.00001).

### 3.6 Erythema

**FIGURE 7:**
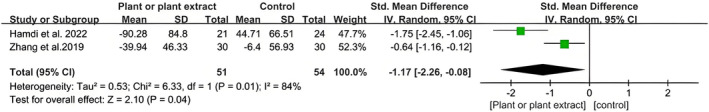
A forest plot derived from a meta‐analysis of these two trials, illustrating the difference in erythema between the intervention and placebo groups.

A total of 105 participants in two studies reported erythema. In one study, a single plant extract was utilized [37], while a variety of herbal extracts were used in the other [38]. The Mexameter was the standard measurement tool used in both studies, with measurements taken on the cheek in one study and on the periocular area in the other (Figure 7).

The analysis revealed a significant statistical difference in erythema associated with the use of plant extracts, as evidenced by the total effect size (SMD = −1.17, 95% CI: −2.26 to −0.08, *p* = 0.04). However, the high heterogeneity (*I* ^2^ = 84%) suggests variability, which may be attributed to the limited number of included studies or differences in the types and quantities of extracts used.

### 3.7 TEWL

**FIGURE 8:**
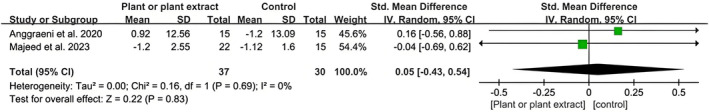
A forest plot derived from a meta‐analysis of these two trials, illustrating the difference in TEWL between the intervention and placebo groups.

Two studies, including 142 participants, reported on transepidermal water loss (TEWL). The product in each study contained only a single plant extract, and the Cutometer dual MP‐580 was employed for measurement. In one study, the dorsum of the hand was measured [39], while in the other, the cheek was assessed (Figure 8; [33]).

No statistically significant differences were observed between plant extracts and placebo (SMD 0.05; 95% CI −0.43 to 0.54, *p* = 0.83), indicating that the plant products did not reduce TEWL.

## 4 Discussion

This paper included eight studies with a total of 396 subjects to conclude whether the use of plants or plant extracts affects five biomarkers of skin aging: Melanin, erythema, skin hydration, skin elasticity, and TEWL. Despite the limited number of samples and studies, the anti‐aging benefits of the topical application of botanicals or botanical extracts had not been previously summarized in the relevant literature. Therefore, this paper provides a comprehensive analysis of the effects of topical botanical preparations on skin aging.

Plant extracts contain potent antioxidants, such as saponins, flavonoids, quercetin, and polyphenols [40, 41, 42], which help maintain the skin's youthful appearance. According to this meta‐analysis, plant preparations are effective in increasing R2 within a treatment duration of ≤ 8 weeks and in decreasing melanin and erythema. However, they have no discernible impact on increasing skin hydration, R5, and R7, or on decreasing TEWL. The skin is composed of three layers: the epidermis, dermis, and subcutaneous tissue [43]. The mechanisms and manifestations of cutaneous aging vary across different stages of the aging process. The primary cause of dry skin is the deficiency of natural moisturizing factors in keratinocytes, which include key components such as amino acids (AAs), trans‐urocanic acid, pyrrolidone carboxylic acid, lactate, and filaggrin—markers of terminal differentiation of the epidermis [44, 45], as well as a decrease in hyaluronic acid (HA) in the dermis [46]. The skin hydration effects of plant extracts have been reported in several studies, involving the upregulation of MAPK, AP‐1, and Akt/PI3K signaling proteins associated with cell proliferation, as well as the presence of moisturizing factors or active ingredients that can increase AQP3 levels. Additionally, some plant extracts can enhance sebum production and promote hydration in keratinocytes by broadly reactivating the production of skin lipids on a broad scale, effectively increasing skin moisture content while reducing the appearance of wrinkles [47, 48, 49]. The dermis consists of collagen‐rich connective tissue [50], with collagen and elastin fibers as its main components [51]. A decrease in the collagen and protofibril content of the dermis, along with an upregulation of fibroblast‐derived elastase activity, can lead to reduced skin elasticity and increased susceptibility to wrinkle formation [52]. The presence of polyphenols in plants enhances the deposition of elastin and collagen in the extracellular matrix, thereby preventing the gradual loss of these proteins [53]. Abnormal pigmentation is a common symptom of skin aging, primarily driven by the synthesis, transfer, and degradation of melanin in melanocytes. This process is influenced by various factors, including oxidative stress [54, 55]. Findings from multiple studies have demonstrated that plant extracts exert inhibitory effects on melanin synthesis by suppressing the transcription of tyrosinase‐related protein [56]. Additionally, other extracts inhibit tyrosinase activity and reduce oxidative stress, further decreasing melanin production [57, 58]. The content of plant extracts, the extraction and production processes, the type of product, and other factors all influence their effectiveness. Additionally, individual variations in sebum secretion categorize skin into different types [59]. There are also differences in transdermal absorption among different types of products [60]. One study showed that a plant extract emulsion is significantly more effective in improving skin hydration and epidermal barrier function than a hydrogel [61]. As a result, individuals should select the type of cosmetic product that best matches their skin type. Although this paper focuses on only two categories of cosmetics, the plants or plant extracts used have not been associated with any adverse events, suggesting they are safe for topical application. However, further research is necessary to confirm their mechanisms of action.

However, this literature has several limitations: (1) Most clinical studies are self‐controlled trials, resulting in fewer randomized controlled trials being included, which has led to a smaller pool of literature in this paper. Additionally, some studies have small sample sizes, with the smallest control and experimental groups comprising only 10 subjects each. (2) The influence of participants' diet and lifestyle choices on the study results cannot be entirely excluded. (3) There are many different types of plants, each with its unique composition and complex mechanisms of action that are not extensively covered in this work.

The majority of the included studies did not report any adverse events, indicating that the use of plant‐based products is generally safe.

## 5 Conclusion

Thus, while plants or plant extracts significantly improved some markers related to skin aging, notable improvements were not observed in other areas, particularly in aspects of skin hydration and TEWL. Nevertheless, this research may represent the most comprehensive study to date on the safety and efficacy of the topical use of plant products in treating skin aging. Further exploration is needed to determine the extent to which plants or plant extracts affect skin aging, including the depth of wrinkles and the minimal erythema dose (MED) associated with skin aging, which have not yet been addressed. Such studies should involve large sample sizes and high‐quality methodologies. We hope that as attention to skin aging continues to grow, more trials will be conducted, and more data will emerge to support the beneficial effects of plants and plant extracts on markers associated with skin aging. Consequently, more effective and safer plant extract products will enter the market to improve skin aging.

## Author Contributions

Z.C. and J.F. wrote the original draft and contributed to formal analysis and investigation. F.C. contributed to supervision, manuscript drafting, and revision. H.L. was responsible for data curation and contributed to writing, review, and editing. Q.D. provided supervision and contributed to writing, review, and editing. All authors have read and approved the final manuscript.

## Ethics Statement

The authors have nothing to report.

## Conflicts of Interest

The authors declare no conflicts of interest.

## Supporting information


Data S1.

## Data Availability

The data that support the findings of this study are available from the corresponding author upon reasonable request.

## References

[1] C. H. Huang , S. C. Chen , Y. C. Wang , C. F. Wang , C. H. Hung , and S. S. Lee , “Detrimental Correlation Between Air Pollution With Skin Aging in Taiwan Population,” Medicine 101, no. 31 (2022): e29380, 10.1097/md.0000000000029380. 35945750 PMC9351911

[2] Y. R. Helfrich , D. L. Sachs , and J. J. Voorhees , “Overview of Skin Aging and Photoaging,” Dermatology Nursing 20, no. 3 (2008): 177–183. 18649702

[3] J. Y. Ng and F. T. Chew , “A Systematic Review of Skin Ageing Genes: Gene Pleiotropy and Genes on the Chromosomal Band 16q24.3 May Drive Skin Ageing,” Scientific Reports 12, no. 1 (2022): 13099, 10.1038/s41598-022-17443-1. 35907981 PMC9338925

[4] M. P. Brincat , Y. M. Baron , and R. Galea , “Estrogens and the Skin,” Climacteric: The Journal of the International Menopause Society 8, no. 2 (2005): 110–123, 10.1080/13697130500118100. 16096167

[5] M. A. Farage , K. W. Miller , P. Elsner , and H. I. Maibach , “Functional and Physiological Characteristics of the Aging Skin,” Aging Clinical and Experimental Research 20, no. 3 (2008): 195–200, 10.1007/bf03324769. 18594185

[6] M. D. Howell , B. E. Kim , P. Gao , et al., “Cytokine Modulation of Atopic Dermatitis Filaggrin Skin Expression,” Journal of Allergy and Clinical Immunology 120, no. 1 (2007): 150–155, 10.1016/j.jaci.2007.04.031. 17512043 PMC2669594

[7] T. M. Ansary , M. R. Hossain , K. Kamiya , M. Komine , and M. Ohtsuki , “Inflammatory Molecules Associated With Ultraviolet Radiation‐Mediated Skin Aging,” International Journal of Molecular Sciences 22, no. 8 (2021): 3974, 10.3390/ijms22083974. 33921444 PMC8069861

[8] Y. Gu , J. Han , C. Jiang , and Y. Zhang , “Biomarkers, Oxidative Stress and Autophagy in Skin Aging,” Ageing Research Reviews 59 (2020): 101036, 10.1016/j.arr.2020.101036. 32105850

[9] S. Moriwaki and Y. Takahashi , “Photoaging and DNA Repair,” Journal of Dermatological Science 50, no. 3 (2008): 169–176, 10.1016/j.jdermsci.2007.08.011. 17920816

[10] W. Zheng , H. Li , Y. Go , X. H. F. Chan , Q. Huang , and J. Wu , “Research Advances on the Damage Mechanism of Skin Glycation and Related Inhibitors,” Nutrients 14, no. 21 (2022): 4588, 10.3390/nu14214588. 36364850 PMC9655929

[11] J. Duan , E. N. Greenberg , S. S. Karri , and B. Andersen , “The Circadian Clock and Diseases of the Skin,” FEBS Letters 595, no. 19 (2021): 2413–2436, 10.1002/1873-3468.14192. 34535902 PMC8515909

[12] L. Shao , S. Jiang , Y. Li , et al., “Regular Late Bedtime Significantly Affects the Skin Physiological Characteristics and Skin Bacterial Microbiome,” Clinical, Cosmetic and Investigational Dermatology 15 (2022): 1051–1063, 10.2147/ccid.S364542. 35698548 PMC9188400

[13] R. Agrawal , A. Hu , and W. B. Bollag , “The Skin and Inflamm‐Aging,” Biology 12, no. 11 (2023): 1396, 10.3390/biology12111396. 37997995 PMC10669244

[14] K. J. Trouba , H. K. Hamadeh , R. P. Amin , and D. R. Germolec , “Oxidative Stress and Its Role in Skin Disease,” Antioxidants and Redox Signaling 4, no. 4 (2002): 665–673, 10.1089/15230860260220175. 12230879

[15] H. Hashizume , “Skin aging and dry skin,” Journal of Dermatology 31, no. 8 (2004): 603–609, 10.1111/j.1346-8138.2004.tb00565.x. 15492432

[16] K. Grice , H. Sattar , M. Sharratt , and H. Baker , “Skin Temperature and Transepidermal Water Loss,” Journal of Investigative Dermatology 57, no. 2 (1971): 108–110, 10.1111/1523-1747.ep12349617. 5097581

[17] K. A. Hwang , B. R. Yi , and K. C. Choi , “Molecular Mechanisms and In Vivo Mouse Models of Skin Aging Associated With Dermal Matrix Alterations,” Laboratory Animal Research 27, no. 1 (2011): 1–8, 10.5625/lar.2011.27.1.1. 21826153 PMC3145984

[18] A. Gonzalez‐Bravo , T. Montero‐Vilchez , S. Arias‐Santiago , and A. Buendia‐Eisman , “The Effect of Sunscreens on the Skin Barrier,” Life 12, no. 12 (2022): 2083, 10.3390/life12122083. 36556448 PMC9784273

[19] L. P. Ma , M. M. Liu , F. Liu , et al., “Melatonin Inhibits Senescence‐Associated Melanin Pigmentation Through the p53‐TYR Pathway in Human Primary Melanocytes and the Skin of C57BL/6 J Mice After UVB Irradiation,” Journal of Molecular Medicine 101, no. 5 (2023): 581–593, 10.1007/s00109-023-02301-y. 37032347 PMC10163137

[20] P. Wattanakrai and K. Nimmannitya , “A Randomized, Double‐Blind, Split‐Face Study of Topical Silymarin vs 2% Hydroquinone Cream in Melasmas,” Journal of Drugs in Dermatology: JDD 21, no. 12 (2022): 1304–1310, 10.36849/jdd.6491. 36468967

[21] M. Zasada , E. Budzisz , and A. Erkiert‐Polguj , “A Clinical Anti‐Ageing Comparative Study of 0.3 and 0.5% Retinol Serums: A Clinically Controlled Trial,” Skin Pharmacology and Physiology 33, no. 2 (2020): 102–116, 10.1159/000508168. 32428912

[22] X. He , F. Wan , W. Su , and W. Xie , “Research Progress on Skin Aging and Active Ingredients,” Molecules 28, no. 14 (2023): 5556, 10.3390/molecules28145556. 37513428 PMC10385838

[23] M. Michalak , “Plant Extracts as Skin Care and Therapeutic Agents,” International Journal of Molecular Sciences 24, no. 20 (2023): 15444, 10.3390/ijms242015444. 37895122 PMC10607442

[24] Y. Wang , Z. Tian , S. Huang , and N. Dang , “ *Tripterygium wilfordii Hook. F*. and Its Extracts for Psoriasis: Efficacy and Mechanism,” Drug Design, Development and Therapy 17 (2023): 3767–3781, 10.2147/dddt.S439534. 38144417 PMC10749103

[25] J. A. Ansong , E. Asante , R. Johnson , et al., “Formulation and Evaluation of Herbal‐Based Antiacne Gel Preparations,” BioMed Research International 2023 (2023): 7838299, 10.1155/2023/7838299. 38146392 PMC10749724

[26] M. S. Ferreira , M. C. Magalhães , R. Oliveira , J. M. Sousa‐Lobo , and I. F. Almeida , “Trends in the Use of Botanicals in Anti‐Aging Cosmetics,” Molecules 26, no. 12 (2021): 3584, 10.3390/molecules26123584. 34208257 PMC8230945

[27] Y. R. Song , W. C. Lim , A. Han , et al., “Rose Petal Extract ( *Rosa gallica* ) Exerts Skin Whitening and Anti‐Skin Wrinkle Effects,” Journal of Medicinal Food 23, no. 8 (2020): 870–878, 10.1089/jmf.2020.4705. 32609563

[28] S. Smiljanic , C. Messaraa , V. Lafon‐Kolb , et al., “ *Betula alba* Bark Extract and *Empetrum nigrum* Fruit Juice, a Natural Alternative to Niacinamide for Skin Barrier Benefits,” International Journal of Molecular Sciences 23, no. 20 (2022): 12507, 10.3390/ijms232012507. 36293365 PMC9604162

[29] D. Moher , A. Liberati , J. Tetzlaff , and D. G. Altman , “Preferred Reporting Items for Systematic Reviews and Meta‐Analyses: The PRISMA Statement,” Open Medicine 3, no. 3 (2009): e123–e130. 21603045 PMC3090117

[30] J. P. Higgins , D. G. Altman , P. C. Gøtzsche , et al., “The Cochrane Collaboration's Tool for Assessing Risk of Bias in Randomised Trials,” BMJ 343 (2011): d5928, 10.1136/bmj.d5928. 22008217 PMC3196245

[31] J. P. Higgins , S. G. Thompson , J. J. Deeks , and D. G. Altman , “Measuring Inconsistency in Meta‐Analyses,” BMJ 327, no. 7414 (2003): 557, 10.1136/bmj.327.7414.557. 12958120 PMC192859

[32] J. Fabrowska , A. Kapuscinska , B. Leska , K. Feliksik‐Skrobich , and I. Nowak , “In Vivo Studies and Stability Study of *Cladophora Glomerata* Extract as a Cosmetic Active Ingredient,” Acta Poloniae Pharmaceutica 74, no. 2 (2017): 633–641. 29624269

[33] M. Majeed , K. Nagabhushanam , S. Paulose , H. R. Rajalakshmi , and L. Mundkur , “A Randomized Double‐Blind, Placebo‐Controlled Study to Evaluate the Anti‐Skin‐Aging Effect of LactoSporin—The Extracellular Metabolite From *Bacillus coagulans* (Weizmannia Coagulans) MTCC 5856 in Healthy Female Volunteers,” Clinical, Cosmetic and Investigational Dermatology 16 (2023): 769–782, 10.2147/ccid.S403418. 37016604 PMC10066892

[34] I. Pagin , S. Togni , G. Maramaldi , and L. Giacomelli , “Anti‐Aging Effects of a Novel Sericoside 0.5% Cream in Reducing Skin Wrinkles and Ameliorating Skin Texture,” Experimental Dermatology 18 (2016): 183–186.

[35] S. S. Roh , I. Choi , H. M. Kim , et al., “Clinical Efficacy of Herbal Extract Cream on the Skin Hydration, Elasticity, Thickness, and Dermis Density for Aged Skin: A Randomized Controlled Double‐Blind Study,” Journal of Cosmetic Dermatology 18, no. 5 (2019): 1389–1394, 10.1111/jocd.12846. 30636339

[36] M. Morag , J. Nawrot , I. Siatkowski , et al., “A Double‐Blind, Placebo‐Controlled Randomized Trial of Serratulae Quinquefoliae Folium, a New Source of β‐Arbutin, in Selected Skin Hyperpigmentations,” Journal of Cosmetic Dermatology 14, no. 3 (2015): 185–190, 10.1111/jocd.12147. 26119285

[37] H. Hamdi , L. Shirbeigi , M. Rahimzadeh , et al., “Evaluation of the Effect of *Artemisia Absinthium* L. Eye‐Cream on Infra‐Orbital Dark Circle: A Randomized, Double‐Blind, Placebo‐Controlled Clinical Trial,” Galen Medical Journal 12 (2023): 1–11, 10.31661/gmj.v12i0.2413. PMC11108669 38774851

[38] Q. Zhang , Y. Tu , H. Gu , et al., “A Cream of Herbal Mixture to Improve Melasma,” Journal of Cosmetic Dermatology 18, no. 6 (2019): 1721–1728, 10.1111/jocd.12938. 30980618 PMC6916559

[39] S. Anggraeni , M. A. Umborowati , D. Damayanti , A. Endaryanto , and C. R. S. Prakoeswa , “Role of *Centella asiatica* and Ceramide in Skin Barrier Improvement: A Double Blind Clinical Trial of Indonesian Batik Workers,” Journal of Basic and Clinical Physiology and Pharmacology 32, no. 4 (2021): 589–593, 10.1515/jbcpp-2020-0510. 34214362

[40] H. S. Kim , H. J. Hwang , W. D. Seo , and S. H. Do , “Oat ( *Avena sativa* L.) Sprouts Restore Skin Barrier Function by Modulating the Expression of the Epidermal Differentiation Complex in Models of Skin Irritation,” International Journal of Molecular Sciences 24, no. 24 (2023): 17274, 10.3390/ijms242417274. 38139104 PMC10743458

[41] K. Boonpisuttinant , T. Taka , W. Ruksiriwanich , et al., “Assessment of In Vitro Anti‐Skin Aging Activities of Phyllanthus Indofischeri Bennet Extracts for Dermatological and Aesthetic Applications,” Scientific Reports 13, no. 1 (2023): 18661, 10.1038/s41598-023-45434-3. 37907639 PMC10618208

[42] P. Chuarienthong , N. Lourith , and P. Leelapornpisid , “Clinical Efficacy Comparison of Anti‐Wrinkle Cosmetics Containing Herbal Flavonoids,” International Journal of Cosmetic Science 32, no. 2 (2010): 99–106, 10.1111/j.1468-2494.2010.00522.x. 20412217

[43] E. Russell‐Goldman and G. F. Murphy , “The Pathobiology of Skin Aging: New Insights Into an Old Dilemma,” American Journal of Pathology 190, no. 7 (2020): 1356–1369, 10.1016/j.ajpath.2020.03.007. 32246919 PMC7481755

[44] S. Verdier‐Sévrain and F. Bonté , “Skin Hydration: A Review on Its Molecular Mechanisms,” Journal of Cosmetic Dermatology 6, no. 2 (2007): 75–82, 10.1111/j.1473-2165.2007.00300.x. 17524122

[45] E. Proksch , J. M. Brandner , and J. M. Jensen , “The Skin: An Indispensable Barrier,” Experimental Dermatology 17, no. 12 (2008): 1063–1072, 10.1111/j.1600-0625.2008.00786.x. 19043850

[46] E. Papakonstantinou , M. Roth , and G. Karakiulakis , “Hyaluronic Acid: A Key Molecule in Skin Aging,” Dermato‐Endocrinology 4, no. 3 (2012): 253–258, 10.4161/derm.21923. 23467280 PMC3583886

[47] S. Yoon , M. Kim , S. Shin , et al., “Effect of *Cirsium japonicum* Flower Extract on Skin Aging Induced by Glycation,” Molecules 27, no. 7 (2022): 2093, 10.3390/molecules27072093. 35408493 PMC9000855

[48] M. Dumas , N. S. Sadick , E. Noblesse , et al., “Hydrating Skin by Stimulating Biosynthesis of Aquaporins,” Journal of Drugs in Dermatology: JDD 6, no. 6 Suppl (2007): s20–s24. 17691206

[49] M. De Tollenaere , E. Chapuis , L. Lapierre , et al., “Overall Renewal of Skin Lipids With Vetiver Extract for a Complete Anti‐Ageing Strategy,” International Journal of Cosmetic Science 43, no. 2 (2021): 165–180, 10.1111/ics.12678. 33253416 PMC8246832

[50] S. H. Shin , Y. H. Lee , N. K. Rho , and K. Y. Park , “Skin Aging From Mechanisms to Interventions: Focusing on Dermal Aging,” Frontiers in Physiology 14 (2023): 1195272, 10.3389/fphys.2023.1195272. 37234413 PMC10206231

[51] R. Ganceviciene , A. I. Liakou , A. Theodoridis , E. Makrantonaki , and C. C. Zouboulis , “Skin Anti‐Aging Strategies,” Dermato‐Endocrinology 4, no. 3 (2012): 308–319, 10.4161/derm.22804. 23467476 PMC3583892

[52] G. Imokawa and K. Ishida , “Biological Mechanisms Underlying the Ultraviolet Radiation‐Induced Formation of Skin Wrinkling and Sagging I: Reduced Skin Elasticity, Highly Associated With Enhanced Dermal Elastase Activity, Triggers Wrinkling and Sagging,” International Journal of Molecular Sciences 16, no. 4 (2015): 7753–7775, 10.3390/ijms16047753. 25856675 PMC4425048

[53] A. Chowdhury , N. Nosoudi , S. Karamched , V. Parasaram , and N. Vyavahare , “Polyphenol Treatments Increase Elastin and Collagen Deposition by Human Dermal Fibroblasts; Implications to Improve Skin Health,” Journal of Dermatological Science 102, no. 2 (2021): 94–100, 10.1016/j.jdermsci.2021.03.002. 33766446

[54] S. A. D'Mello , G. J. Finlay , B. C. Baguley , and M. E. Askarian‐Amiri , “Signaling Pathways in Melanogenesis,” International Journal of Molecular Sciences 17, no. 7 (2016): 1144, 10.3390/ijms17071144. 27428965 PMC4964517

[55] A. Y. Lee , “Skin Pigmentation Abnormalities and Their Possible Relationship With Skin Aging,” International Journal of Molecular Sciences 22, no. 7 (2021): 3727, 10.3390/ijms22073727. 33918445 PMC8038212

[56] Y. H. Huang , T. H. Lee , K. J. Chan , F. L. Hsu , Y. C. Wu , and M. H. Lee , “Anemonin Is a Natural Bioactive Compound That Can Regulate Tyrosinase‐Related Proteins and mRNA in Human Melanocytes,” Journal of Dermatological Science 49, no. 2 (2008): 115–123, 10.1016/j.jdermsci.2007.07.008. 17766092

[57] W. Zhu and J. Gao , “The Use of Botanical Extracts as Topical Skin‐Lightening Agents for the Improvement of Skin Pigmentation Disorders,” Journal of Investigative Dermatology Symposium Proceedings 13, no. 1 (2008): 20–24, 10.1038/jidsymp.2008.8. 18369335

[58] R. Jiang , X. H. Xu , K. Wang , et al., “Ethyl Acetate Extract From *Panax ginseng* C.A. Meyer and Its Main Constituents Inhibit α‐Melanocyte‐Stimulating Hormone‐Induced Melanogenesis by Suppressing Oxidative Stress in B16 Mouse Melanoma Cells,” Journal of Ethnopharmacology 208 (2017): 149–156, 10.1016/j.jep.2017.07.004. 28689798

[59] J. Qin , L. Qiao , J. Hu , et al., “New Method for Large‐Scale Facial Skin Sebum Quantification and Skin Type Classification,” Journal of Cosmetic Dermatology 20, no. 2 (2021): 677–683, 10.1111/jocd.13576. 32585060

[60] A. Otto , J. du Plessis , and J. W. Wiechers , “Formulation Effects of Topical Emulsions on Transdermal and Dermal Delivery,” International Journal of Cosmetic Science 31, no. 1 (2009): 1–19, 10.1111/j.1468-2494.2008.00467.x. 19134123

[61] A. Ratz‐Łyko , J. Arct , and K. Pytkowska , “Moisturizing and Antiinflammatory Properties of Cosmetic Formulations Containing *Centella asiatica* Extract,” Indian Journal of Pharmaceutical Sciences 78, no. 1 (2016): 27–33, 10.4103/0250-474x.180247. 27168678 PMC4852572

