# Supplementary material for: Efficacy and Safety of Topical Application of Plant‐Based Products on Skin Aging in Healthy Individuals: A Systematic Review and Meta‐Analysis of Randomized Controlled Trials
Source: J Cosmet Dermatol. 2024 Dec 9;24(2):e16710. doi: 10.1111/jocd.16710 (PMC11845950; doi:10.1111/jocd.16710)

**Supporting Information**

**Table S1**. Search strategy used for this review.

| Database | Search strategy | Number of results |
| --- | --- | --- |
| Pubmed | ((((((((((((((((((((((((((((((((((Plant Extracts[MeSH Terms]) OR (Plants[MeSH Terms])) OR (Phytochemical[MeSH Terms])OR (Plant[Title/Abstract])) OR (Plant Extract[Title/Abstract])) OR (Herbal Medicines[Title/Abstract])) )) OR (Herbal medicine[MeSH Terms])) OR OR (plant[Title/Abstract])) OR (botanical[Title/Abstract])) OR (natural product[Title/Abstract])) OR (Plant Bioactive Compound[Title/Abstract])) OR (Plant Biologically Active Compound[Title/Abstract])) OR (Phytochemical[Title/Abstract])) OR (alga[Title/Abstract])) AND ((((((((((((((((((((((((((((skin aging[MeSH Terms]) OR (Skin Pigmentation[MeSH Terms]) OR (skin care[MeSH Terms])) OR (Hyperpigmentation[MeSH Terms])OR (skin condition[Title/Abstract])) OR (skin barrier[Title/Abstract])) OR (Wrinkle[Title/Abstract])) OR (skin roughness[Title/Abstract])) OR (Aging, Skin[Title/Abstract])) OR (Solar Aging of Skin[Title/Abstract])) OR (Photoaging of Skin[Title/Abstract])) OR (Skin Wrinklings[Title/Abstract])) OR (anti-ageing [Title/Abstract] ))OR (photoaged facial skin[Title/Abstract]))OR (skin health[Title/Abstract]))) OR (Skin Tone[Title/Abstract])) OR (Skin Color[Title/Abstract]OR (photoageing[Title/Abstract]))))))))))))NOT (((Meta-Analysis[Publication Type]) OR (Systematic Review[Publication Type])) OR (Review[Publication Type]))) NOT (mice[MeSH Terms])) NOT (rats[MeSH Terms]) | （979） |
| Embase | Embase  Session Results  .......................................................  No. Query Results Results Date  #9. #7 AND #8  #8. 'randomized controlled trial':de OR 'controlled 2,687,685  clinical trial':de OR 'randomized':ti,ab OR  'placebo':ti,ab OR 'clinical trials' OR  'randomly':ti,ab OR 'clinical study':ti,ab OR  'volunteers':ti,ab  #7. #3 AND #6 5,994  #6. #4 OR #5 118,532  #5. 'aging skin':ab,kw,ti OR 'senile skin':ab,kw,ti 31,350  OR 'skin aging':ab,kw,ti OR 'skin  condition':ab,kw,ti OR 'skin barrier':ab,kw,ti OR  'wrinkle':ab,kw,ti OR 'skin roughness':ab,kw,ti  OR 'aging, skin':ab,kw,ti OR 'solar aging of  skin':ab,kw,ti OR 'photoaging of skin':ab,kw,ti  OR 'skin wrinklings':ab,kw,ti OR  'anti-ageing':ab,kw,ti OR 'photoaged facial  skin':ab,kw,ti OR 'skin health':ab,kw,ti OR 'skin  tone':ab,kw,ti OR 'skin color':ab,kw,ti OR  'photoageing':ab,kw,ti  #4. 'skin pigmentation'/exp OR 'skin pigmentation' OR 91,415  'skin care'/exp OR 'skin care' OR  'hyperpigmentation'/exp OR 'hyperpigmentation'  #3. #1 OR #2 1,750,280  #2. 'plant extract':ab,kw,ti OR 'herbal 610,074  medicines':ab,kw,ti OR 'plant':ab,kw,ti OR  'botanical':ab,kw,ti OR 'natural  product':ab,kw,ti OR 'plant bioactive  compound':ab,kw,ti OR 'plant biologically active  compound':ab,kw,ti OR 'phytochemical':ab,kw,ti OR  'alga':ab,kw,ti  #1. 'plant extract'/exp OR 'plant extract' OR 1,722,382  'plant'/exp OR 'plant' OR 'phytochemical'/exp OR  'phytochemical' OR 'herbal medicine'/exp OR  'herbal medicine' | （1013） |
| Web of science | **(((((((((((((((((((TS=(skin aging)) OR TS=(Epidermis)) OR TS=(skin care)) OR TS=(skin conditions)) OR TS=(skin barrier)) OR TS=(Wrinkle)) OR TS=(skin roughness)) OR TS=(Solar Aging of Skin)) OR TS=(Photoaging of Skin)) OR TS=(Skin Wrinkling)) OR TS=(Skin Wrinklings)) OR TS=(UVB)) OR TS=(anti-ageing)) OR TS=(photoaged facial skin)) OR TS=(skin health)) OR TS=(Skin Pigmentation)) OR TS=(Hyperpigmentation)) OR TS=(Skin Tone)) OR TS=(Skin Color)) AND (((((((((((((TS=(Plant Extracts )) OR TS=(Plants)) OR TS=(Phytochemical )) OR TS=(Plant )) OR TS=(Plant Extract)) OR TS=(Herbal Medicines)) OR TS=(Herbal medicine )) OR TS=(botanical )) OR TS=(natural product)) OR TS=(Plant Bioactive Compound)) OR TS=(Plant Biologically Active Compound)) OR TS=(Phytochemical )) OR TS=(alga))**AND(((((((TS=(randomized controlled trial)) OR TS=(controlled clinical trial)) OR TS=(randomized)) OR TS=(placebo)) OR TS=(clinical trials)) OR TS=(randomly)) OR TS=(clinical study)) OR TS=(volunteers) NOT((TS=(Meta-Analysis)) OR TS=(Systematic Review)) OR TS=(Review)NOT(TS=(mice)) OR TS=(rats) | （662） |
| Cochrane | #1 MeSH descriptor: [Skin Pigmentation] explode all trees 435  #2 MeSH descriptor: [Skin Aging] explode all trees 1049  #3 MeSH descriptor: [Hyperpigmentation] explode all trees 774  #4 MeSH descriptor: [Skin Care] explode all trees 856  #5 (skin condition):ti,ab,kw OR (skin barrier):ti,ab,kw OR (Wrinkle):ti,ab,kw OR (skin roughness):ti,ab,kw OR (Aging, Skin):ti,ab,kw OR (Solar Aging of Skin):ti,ab,kw OR (Photoaging of Skin):ti,ab,kw OR (Skin Wrinklings):ti,ab,kw OR (anti-ageing ):ti,ab,kw OR (photoaged facial skin):ti,ab,kw OR (skin health):ti,ab,kw OR (Skin Tone):ti,ab,kw OR (Skin Color):ti,ab,kw OR (photoageing):ti,ab,kw 25972  #6 #1 OR #2 OR #3 OR #4 OR #5 27207  #7 Plant Extracts 5885  #8 MeSH descriptor: [Plant Extracts] explode all trees 9867  #9 MeSH descriptor: [Plants] explode all trees 3564  #10 MeSH descriptor: [Phytochemicals] explode all trees 517  #11 MeSH descriptor: [Herbal Medicine] explode all trees 243  #12 (Plant):ti,ab,kw OR (Plant Extract):ti,ab,kw OR (Herbal Medicines):ti,ab,kw OR (plant):ti,ab,kw OR (botanical):ti,ab,kw OR (natural product):ti,ab,kw OR (Plant Bioactive Compound):ti,ab,kw OR (Plant Biologically Active Compound):ti,ab,kw OR (Phytochemical):ti,ab,kw OR (alga):ti,ab,kw 18277  #13 #7 OR #8 OR #9 OR #10 OR #11 OR #12 25629  #14 #6 AND #13 799 | (799) |

**Table S2.** Full details of all studies included in systematic review.

| **Study Location** | **Study Population** | | | **Intervention** | **Control** | **Formulation** | **Study Duration** | **Outcome** | | **Adverse Effects** |
| --- | --- | --- | --- | --- | --- | --- | --- | --- | --- | --- |
|  | **Sample size, sex** | **Age, years** | **Health Condition** | **Contents, daily dose** | **Contents, daily dose** |  |  | **Meaurement Instrument** | **Parameter (Measuring Sites)** |  |
| FABROWSKA 2017  Poland | n=20  Unclear | 20-50 | Healthy different  age profile | Cladophora glomerata extract | placebo | Emulsion | 4w | Corneometer CM 825, Cutometer® Dual MPA 580 | Arm | AE=0 |
| Morag 2015  Poland | n=102  100% F | 26-55 | Healthy,  two types of skin  hyperpigmentation: melasma and lentigo solaris. | 100mg  leaves of five-leaf serratula | placebo | Cream | 8w | Mexametrâ | Cheek | NR |
| Majeed 2023  India | n=56  100%F | 43.83±6.61 | Healthy;visible fine lines  And  wrinkles;  Fitzpatrick  skin type III  to V, | Extract of B. coagulans (W. coagulans) | carbomer sorbitan  stearate(and) sucrose Cocoate | Cream | 12w | Cutometer MPA580  Corneometer CM 825, | Cheek | AE=0 |
| PAGIN 2016  Italy | n=40;  Unclear | 35-65 | Healthy,, presenting  wrinkles, lack of skin elasticity, dull skin complexion, eyelid  bags and/or dark circles | sericoside-based formulation | Placebo cream | Cream | 30D | Derma Top Blue  Minolta Chromameter CR-200  MPA® 580 | periocular | NR |
| Zhang 2019  China | n=60  Unclear | 18-59 | Healthy,Men and women with melasma  Fitzpatrick skin types III and IV | China camellia  sanchi prinsepia utilis oil and portulaca oleracea | Placebo cream | Cream | 12W | Mexameter | Cheek | NR |
| Roh 2018  Korea | n=43  100%F | 40-59 | Healthy | 0.5g of water methyl propanediol glycyrrhiza glabra (licorice) root extract  Angelica gigas root extract Prunus persica (peach) kernel extract | Water, cyclohexasiloxane, hydrogenated polydecene, dipropylene  glycol, | Cream | 8w | Corneometer CM 825, Cutometer® Dual MPA 580 | Cheek | NR |
| Hamdi 2022  Iran | n=45  Unclear | 18-65 | Healthy， had dark circles under their eyes | aqueous extract of A.absinthium i | Placebo | cream | 60 d | Mexameter® | periocular | NR |
| Anggraeni 2020  Indonesia | n=30  100% F | 15-50 | Healthy， skin  dryness | asiatica 2% in  ceramide based cream) | ceramide 5% cream | cream | 4w | Cutometer dual MP-580 | Hand | NR |

F=Female

W=Week

AE = adverse events

NR = not reported

**Figure S1：**Summary of risk of bias: evaluate the authors' assessments of each risk of bias domain for every study that is included.


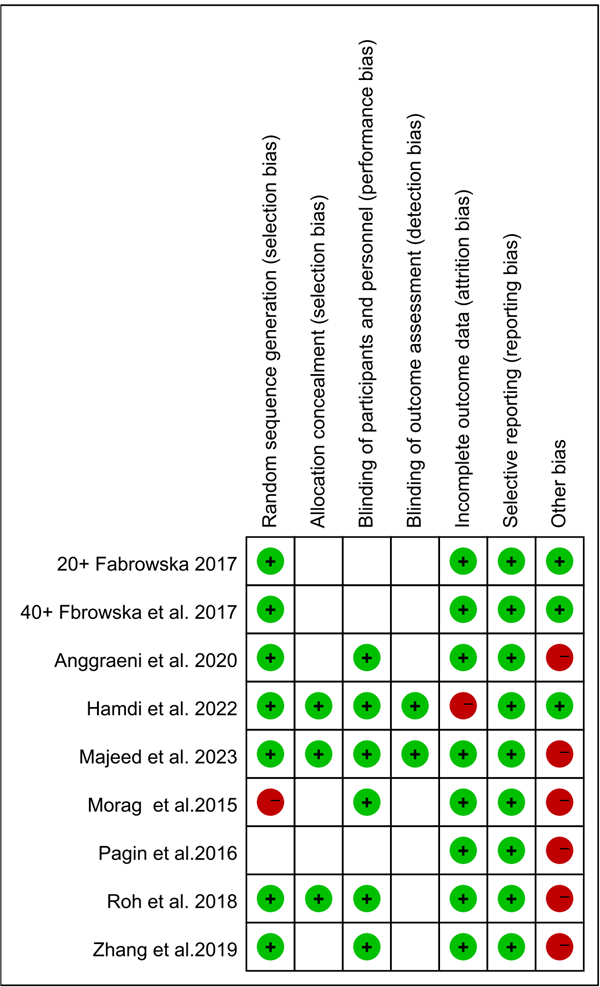


**Figure S2**| Plant or plant extract vs. placebo in a forest plot for R2 (SMD) Confidence interval (CI) for Roh 2018 after exclusion.


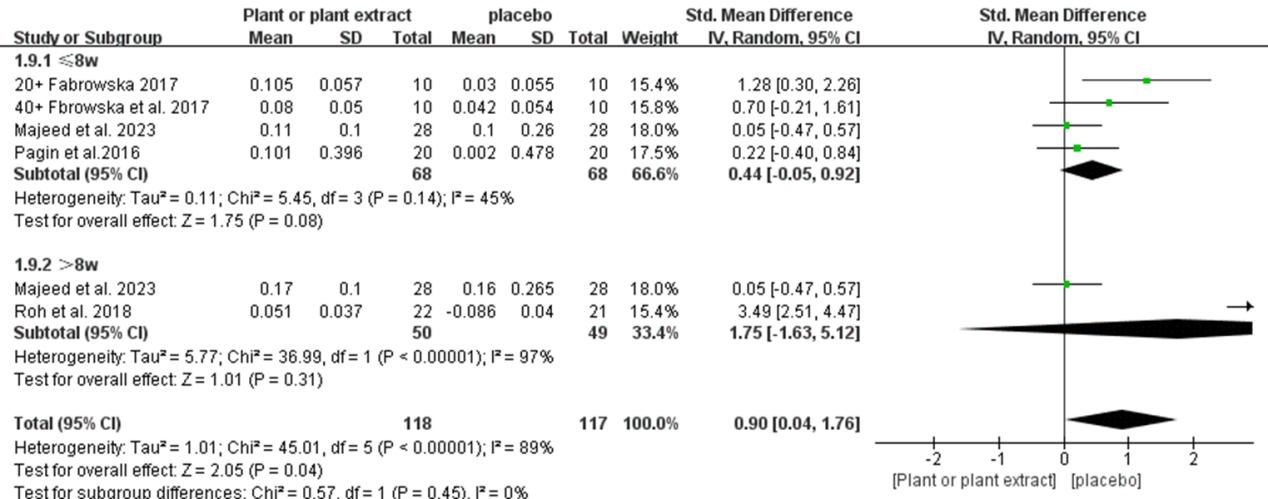


**Figure S3**| Plant or plant extract vs. placebo in a forest plot for skin hydration (SMD) Confidence interval (CI) for Majeed 2023 after exclusion.


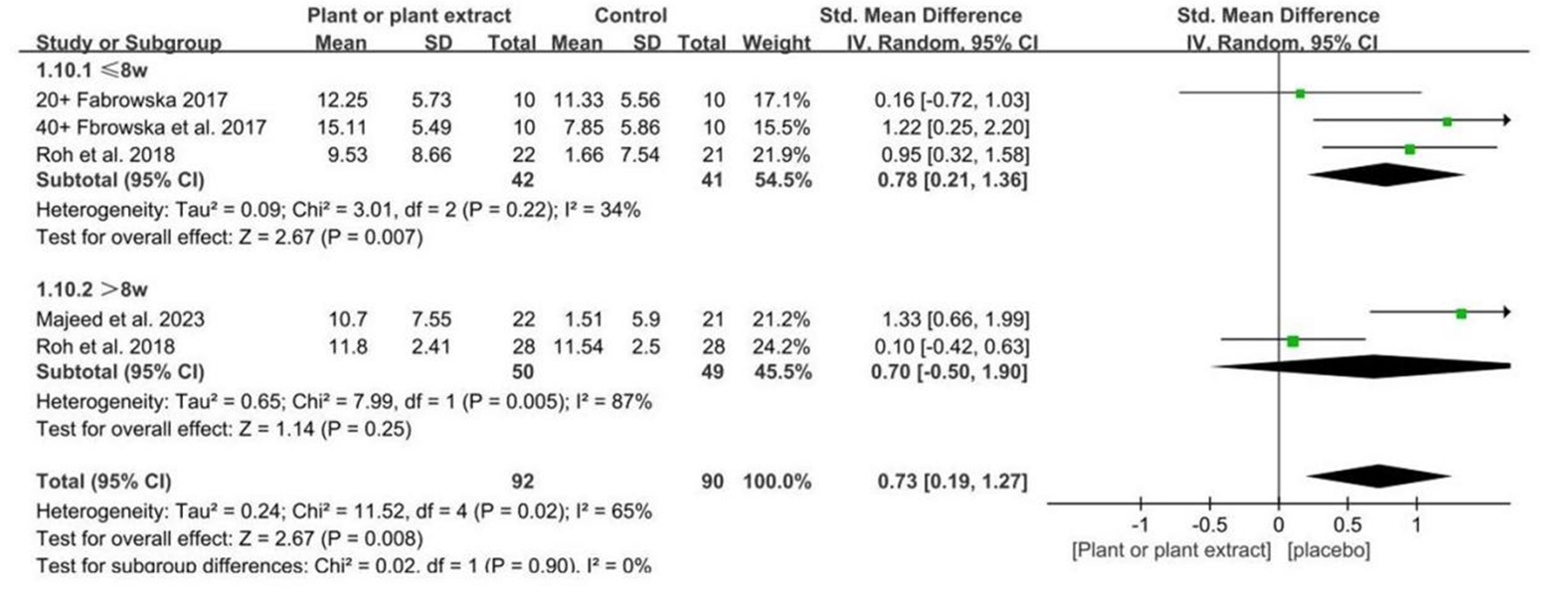

Supplement: Supplementary file 1 — Data S1. [file JOCD-24-e16710-s001.docx]
